# Beyond Adult Stem Cells: Dedifferentiation as a Unifying Mechanism Underlying Regeneration in Invertebrate Deuterostomes

**DOI:** 10.3389/fcell.2020.587320

**Published:** 2020-10-20

**Authors:** Cinzia Ferrario, Michela Sugni, Ildiko M. L. Somorjai, Loriano Ballarin

**Affiliations:** ^1^Department of Environmental Science and Policy, University of Milan, Milan, Italy; ^2^Center for Complexity and Biosystems, Department of Physics, University of Milan, Milan, Italy; ^3^GAIA 2050 Center, Department of Environmental Science and Policy, University of Milan, Milan, Italy; ^4^The Willie Russel Laboratories, Biomedical Sciences Research Complex, North Haugh, University of St Andrews, St Andrews, United Kingdom; ^5^Department of Biology, University of Padua, Padua, Italy

**Keywords:** adult invertebrate deuterostomes, dedifferentiation, progenitor cells, stem cells, regeneration

## Abstract

The diversity of regenerative phenomena seen in adult metazoans, as well as their underlying mechanistic bases, are still far from being comprehensively understood. Reviewing both ultrastructural and molecular data, the present work aims to showcase the increasing relevance of invertebrate deuterostomes, i.e., echinoderms, hemichordates, cephalochordates and tunicates, as invaluable models to study cellular aspects of adult regeneration. Our comparative approach suggests a fundamental contribution of local dedifferentiation -rather than mobilization of resident undifferentiated stem cells- as an important cellular mechanism contributing to regeneration in these groups. Thus, elucidating the cellular origins, recruitment and fate of cells, as well as the molecular signals underpinning tissue regrowth in regeneration-competent deuterostomes, will provide the foundation for future research in tackling the relatively limited regenerative abilities of vertebrates, with clear applications in regenerative medicine.

## Introduction

Since the time of Aristotle, regeneration has been one of the most fascinating and perplexing biological phenomena to explain, challenging, as it does, the common dogma of irreversibility of ontogenetic processes. After an initial period of descriptive studies (Spallanzani, 1768; Morgan, 1901; Maienschein, 2011), more recent research has begun to delve into the deeper and more complex mechanistic problems underlying the regenerative process. In particular, where new cells come from -and how they acquire their correct committed fate- to achieve a successful regenerative outcome are two of the most pressing issues faced, and yet they still need to be fully clarified.

In attempting to characterize and classify the origins of the cells contributing to the new regenerate, two broad regeneration modalities have classically been distinguished: i) *morphallaxis*, or regeneration relying mainly on the remodeling of pre-existing cells and tissues; and ii) regeneration proceeding through the formation of a blastema, also known as *epimorphosis*. In the latter, a mass of undifferentiated cells of mesenchymal origin and enveloped by an epithelial layer is formed at the amputation site by recruitment of cells and their extensive proliferation (for high quality illustrations depicting these processes see for example Sánchez Alvarado and Tsonis, 2006; Gentile et al., 2011). These definitions were proposed when no detailed analyses of regenerative phenomena were possible at the cellular and molecular level (Morgan, 1901). In some cases, the original terms have even been adapted to better fit local case-studies, such that agreement on any clear and unequivocal definition appears to be lacking. However, it is now evident that these two modalities lie along a spectrum, frequently difficult to distinguish in practical terms, and often coexist (Candia Carnevali, 2006; Agata et al., 2007). In an effort to reconcile some of the difficulties caused by these terms, an alternative perspective unifying the two principles -and based on positional identity of cells- was proposed, the so-called “distalization-intercalation” model (Agata et al., 2007). According to this model, during regeneration the most distal cells are replaced first, going on to act as an “organizer” and new signaling center for patterning of the intervening tissues. Cross-talk between this distal element and the old stump tissues induces reorganization of positional information so that the new tissues are regenerated between these two positional extremities. Cells and tissues of the distal entity vary depending on the model system in question, and include for instance the wound epidermis formed during limb regeneration in urodeles or the distal tip cells of the blastema in bisected planaria. This model can be even considered a “universal developmental model” not only applicable to regeneration but also to embryogenesis (Ben Khadra et al., 2018b). While we fully agree with this modern perspective, in the present review we still sometimes use the original terminology referring to epimorphosis and morphallaxis in order to faithfully represent specific cellular processes described in earlier work.

Regardless of the underlying mechanism used, the ability to regenerate missing body parts relies on the availability of a source of multipotent/pluripotent cells. These can either be undifferentiated adult stem cells (ASCs), or they can derive from dedifferentiation/redifferentiation processes (Sánchez Alvarado, 2000; see glossary). Typical examples of ASCs include sponge archeocytes (Funayama, 2018), cnidarian interstitial cells (Frank et al., 2009), flatworm neoblasts (De Mulder et al., 2009; Salvetti and Rossi, 2019), annelid teloblasts (Sugio et al., 2012; Gazave et al., 2013) and some vertebrate lineage-restricted stem cells [e.g., muscle satellite cells, neural stem cells, etc. (Marques et al., 2019)]. However, a deeper understanding of the relative contributions of ASCs and dedifferentiation during animal regeneration is still lacking, and the roles of cell proliferation dynamics and the microenvironment/extracellular matrix (“niches”) (García-Arrarás, 2018; Lai and Aboobaker, 2018) in directing different regenerative outcomes require more extensive research.

Although ultrastructural and molecular analyses can provide important insights into the temporal and spatial distribution of different cytotypes in regenerating tissues, only cell tracking studies can definitively clarify the actual origin and fate of cells recruited to restore functional body parts. At present this type of study has been performed only in a very limited number of regeneration-competent animal models, chosen for their long history of regeneration research or their genetic tractability. Currently, this includes a few vertebrate systems, e.g., urodele and anuran amphibians (Brito, 2018; Gross, 2018; Aztekin et al., 2019), and zebrafish (Pfefferli and Jaźwińska, 2015), and a handful of invertebrates, such as *Hydra* (Bosch, 2007) and planarians (Pellettieri, 2019; Rossi and Salvetti, 2019). However, these models comprise only a subset of the diversity of regenerative phenomena present in the animal kingdom, and are often difficult to compare due to large evolutionary distances. Understanding how lineage and cell fate decisions are made through a comparative approach in a wider organismal diversity, therefore, still represents one of the main challenges for the scientific community.

Beyond how and why animals regenerate (Bely and Nyberg, 2010), it is critical to understand the nature of the constraints impeding regeneration (Bely, 2010). With the few notable exceptions already mentioned, vertebrates generally display limited regeneration competence, restricted at best to some organs or tissues (e.g., fins, cornea, liver, epidermis) (Pfefferli and Jaźwińska, 2015; Forbes and Newsome, 2016; Gawronska-Kozak and Bukowska, 2017; Vergara et al., 2018). This is likely related to the appearance of the finely tuned adaptive immune system (Tiozzo and Copley, 2015; Abnave and Ghigo, 2019). Revealing the causes of these limited capabilities is currently one of the most intriguing areas of investigation, and requires an understanding of the mechanisms promoting cell growth and differentiation, tissue homeostasis, aging and senescence. All these processes are of fundamental importance, especially in light of possible applications in the field of human regenerative medicine.

In contrast to vertebrates, invertebrates offer a number of advantages, ranging from (but not limited to) their simpler body organization to their unique regeneration phenomena. These include whole body regeneration (see below), or the presence of unique “stemness” systems, with stem cells spread throughout the body and not necessarily restricted to defined niches (Sköld et al., 2009). In addition, invertebrates continue to reveal unexpected gene regulatory pathways of great interest for regenerative biology (Ballarin et al., 2018).

**FIGURE 1:**
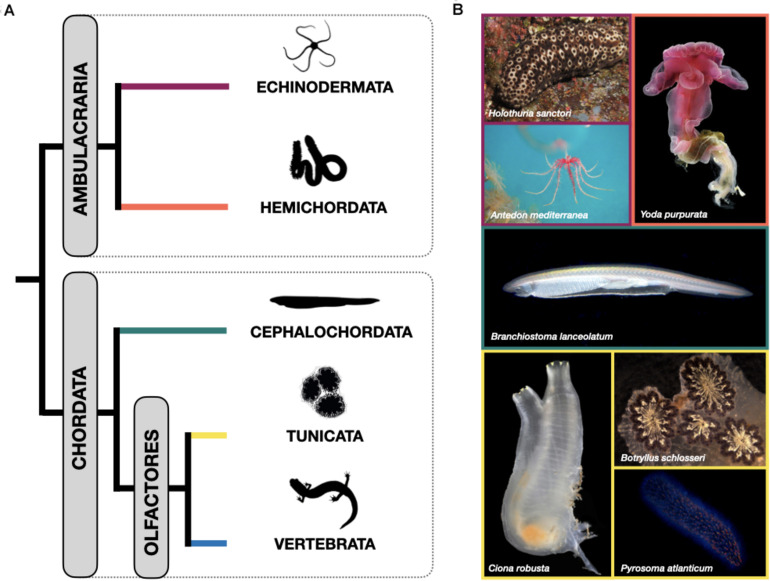
**(A)** Schematic showing the currently accepted phylogenetic relationships among the phyla within the deuterostomes. Echinodermata and Hemichordata are collectively referred to as Ambulacraria. Within the chordates, Cephalochordata are the sister group to Urochordata and Vertebrata, which together comprise the Olfactores. **(B)** Living representatives of the invertebrate deuterostome phyla discussed here. Note the considerable diversity in body plan types even within phyla. Echinodermata: Holothuroidea: *Holothuria sanctori* (credits: Dr Federico Betti, University of Genova), and Crinoidea: *Antedon mediterranea* (credits: Dr Michela Sugni, University of Milan). Hemichordata: Enteropneusta: *Yoda purpurata* (credits: “Smithson Picture 66” by public.resource.org, licensed under CC PDM 1.0). Cephalochordata: *Branchiostoma lanceolatum* (credits: Dr Ildiko Somorjai, University of St Andrews). Tunicata: Ascidiacea: *Ciona robusta* (credits: Dr E.A, Lazo-Wasem, Yale Peabody Museum) and *Botryllus schlosseri* (Dr Loriano Ballarin, University of Padova), and Thaliacea: *Pyrosoma atlanticum* (credits: Dr Alan Deidun, University of Malta).

The invertebrate deuterostomes -which include echinoderms, hemichordates, cephalochordates and tunicates- are considered excellent systems to study regeneration, but are still largely unexplored. Not only do they display a huge range of regenerative potential, with its associated complexity of mechanisms, but their phylogenetic position makes them ideally placed to study the evolution of regenerative abilities, with particular reference to the invertebrate-vertebrate transition (Figure 1A). Therefore, these so-called “emerging” model systems provide a unique opportunity to shed light on the diversity of cell recruitment mechanisms contributing to regeneration in the earliest diverging deuterostomes.

Here, we provide an updated and comprehensive overview of the molecular and cellular basis of adult regeneration in the closest living relatives to vertebrates -the invertebrate deuterostomes- describing presumptive origins and fates of cells contributing to the new tissues. Using both ultrastructural and molecular data, similarities and differences among models are highlighted. Overall, our comparative approach contributes to a deeper understanding of the constraints preventing large scale regeneration in vertebrates, and offers new perspectives to inform this emerging research field.

## Echinodermata

**FIGURE 2:**
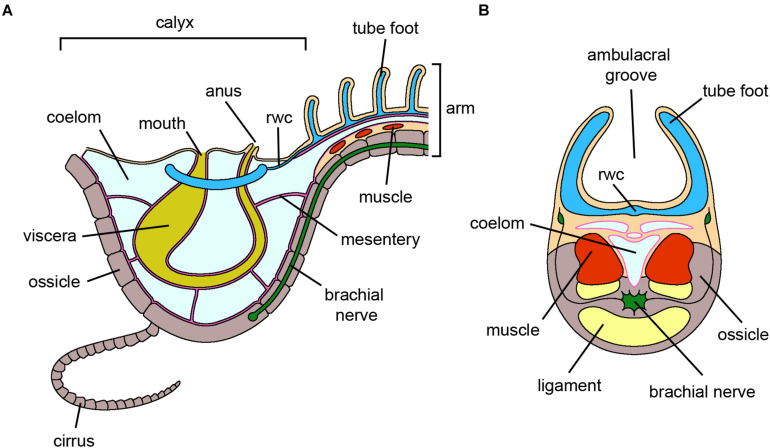
Crinoidea. **(A)** Schematic section through the vertical plane of the calyx and of an arm of an adult crinoid. The oral side, harboring both mouth and anus, faces the water column. The visceral mass is hosted in the calyx and is anchored to the coelomic walls by mesenteries. For simplicity, only one cirrus at the base of the calyx is shown. **(B)** Schematic cross section of an arm of an adult crinoid. The ambulacral groove, including rows of tube feet, faces the water column. Adjacent segments are joined by muscles and ligaments. The brachial nerve longitudinally runs along the arm within the ossicles. For clarity, pinnules and gonads are not shown. *Abbreviations*: rwc-radial water canal. Pink lining represents the coelomic epithelium (somatocoel) (credits: Alessandro Allievi).

**FIGURE 3:**
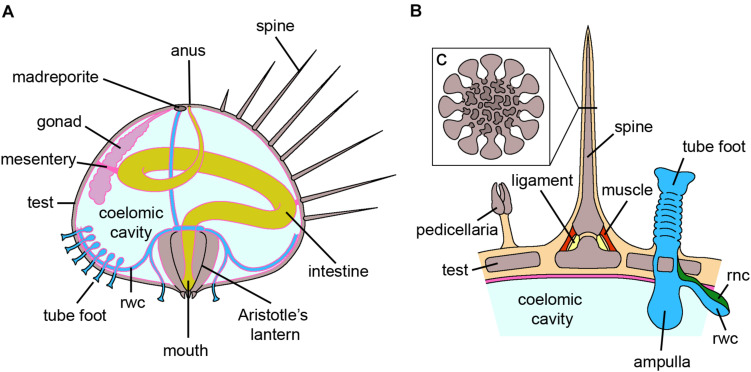
Echinoidea. **(A)** Schematic section through the vertical plane of an adult sea urchin. The oral side, containing the mouth with the Aristotle’s lantern, faces the substrate, whereas the aboral side, including madreporite and anus, faces the water column. The digestive tube is anchored to the internal walls of the test by mesenteries. For clarity, structures that are serially repeated along the test either externally or internally have been only partially shown. **(B)** Schematic longitudinal section of the test where a spine, a tube foot and a pedicellaria are present. The spine is articulated to the test by muscles and ligaments and the tube foot is directly connected to the rwc. **(C)** Insert of **B** showing the schematic cross section of a spine where the inner stereom architecture is visible. *Abbreviations:* rnc-radial nerve cord, rwc-radial water canal. Pink lining represents the coelomic epithelium (credits: Alessandro Allievi).

**FIGURE 4:**
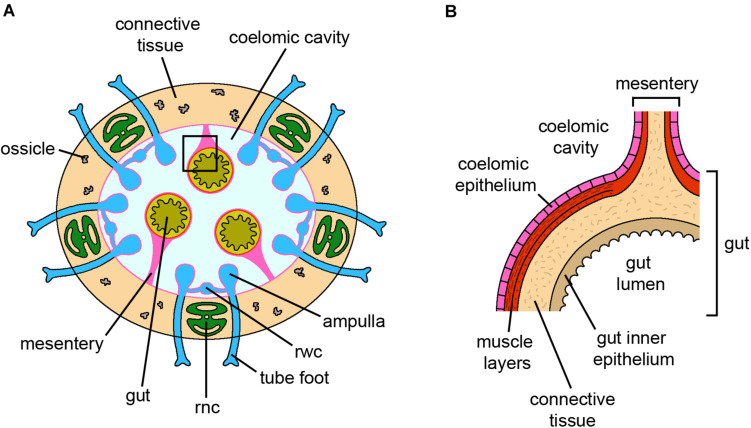
Holothuroidea. **(A)** Schematic cross section of an adult sea cucumber. The body wall is mainly composed of connective tissue with only few small ossicles. The gut is anchored to the coelomic cavity walls by mesenteries. For simplicity, the gonads, located within the coelomic cavity, and the muscle layers of the coelomic cavity wall are not shown. **(B)** Detail of *a* (square) on the gut and the corresponding mesentery. Both structures are lined by coelomic epithelium. *Abbreviations:* rnc-radial nerve cord, rwc-radial water canal. Pink lining represents the coelomic epithelium (credits: Alessandro Allievi).

**FIGURE 5:**
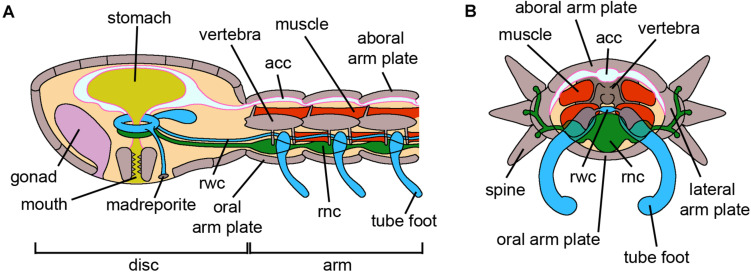
Ophiuroidea. **(A)** Schematic longitudinal section of the disk and an arm of an adult brittle star. The oral side, where the mouth and madreporite are located, faces the substrate. The disk encloses the gonads and the digestive tube, which lacks an anus. The arm is subdivided into serially repeated segments and the inner adjacent vertebrae are articulated by muscles and ligaments. The acc, the rwc and the rnc longitudinally run along the arm. Both disc and arms present skeletal elements called plates, with different names depending on their position. **(B)** Schematic cross section of an arm of an adult brittle star where all structures are visible. Spines are articulated to the lateral arm plates and spinal ganglia are present at their bases. The acc occupies the aboral side of the arm, immediately below the aboral arm plate, and laterally branches near the lateral arm plates. The rnc is the most oral structure above the oral arm plate. *Abbreviations*: acc-aboral coelomic cavity, rnc-radial nerve cord, rwc-radial water canal. Pink lining represents the coelomic epithelium (credits: Alessandro Allievi).

**FIGURE 6:**
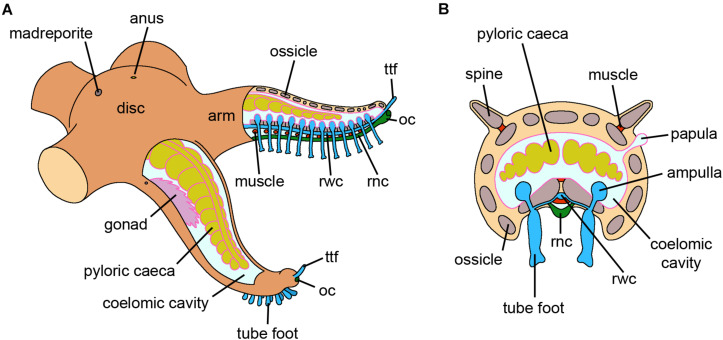
Asteroidea. **(A)** Schematic drawing of an adult starfish where both external and internal anatomy are visible. The aboral side, including the madreporite and the anus, faces the water column. Gonads and pyloric caeca are present within the coelomic cavity. The rwc and the rnc run longitudinally along the arm. The last tube foot of each arm is called the terminal tube foot; the optic cushion, the photoreceptor of the animal, is located orally at its base. **(B)** Schematic cross section of an arm of an adult starfish where all structures are visible. The spines are articulated with the corresponding ossicles of the body wall. Papule, evaginations of the coelomic cavity, are internally lined by coelomic epithelium. The rnc is exposed to the external environment but partially protected by the rows of tube feet. *Abbreviations:* oc-optic cushion, rnc-radial nerve cord, rwc-radial water canal, ttf-terminal tube foot. Pink lining represents the coelomic epithelium (credits: Alessandro Allievi).

Echinoderms are common marine invertebrates and include about 7000 extant species, highly diversified in overall body morphology (Figure 1B; globular, star-shaped, etc.) and divided into five clades: crinoids (sea lilies and feather stars; Figure 2), echinoids (sea urchins and sand dollars; Figure 3), holothuroids (sea cucumbers; Figure 4), ophiuroids (brittle stars; Figure 5) and asteroids (starfish; Figure 6). Members of this phylum display some of the most spectacular regenerative abilities found in the animal kingdom and an impressive diversity of models for studies of regeneration. Regeneration is apparently so common that one could argue it is present in most (if not all) species. Therefore, it is not surprising that they have been used as inspiring biological models for innovative regenerative medicine applications (Di Benedetto et al., 2014a; Ferrario et al., 2017). Irrespective of the life stage or lost body part, representatives from all clades show regenerative potential after both self-induced and traumatic mutilations, and this occurs at the level of tissue, organ or complex body structure (Candia Carnevali, 2006). The most extensive regeneration capabilities are strictly linked with asexual reproduction by fission, as found in representatives of asteroids, ophiuroids and holothuroids (Emson and Wilkie, 1980; McGovern, 2002; Dolmatov, 2014). Some of the best-known examples of regeneration include the formation of a whole animal from a single starfish arm, termed “comet” (Hyman, 1955; Emson and Wilkie, 1980; Mladenov and Burke, 1994; Shibata and Komatsu, 2011; Cortés Rivera et al., 2016); the regrowth of viscera and the nervous system in sea cucumbers (García-Arrarás et al., 1998, 2018); the regeneration of arms after both autotomy and traumatic amputations in starfish, brittle stars and crinoids (Candia Carnevali et al., 1998; Thorndyke et al., 1999; Ben Khadra et al., 2018b); and the regeneration of spines and tests in sea urchins (Dubois and Ameye, 2001; Bonasoro et al., 2004).

Echinoderms are basal deuterostomes, grouped with hemichordates in the clade Ambulacraria, which is the sister group of chordates (Arnone et al., 2015; Figure 1A). Therefore, knowledge of their regenerative processes allows the study of deuterostome regeneration from an evolutionary perspective. Examples of regenerating echinoderms are already present in the fossil record of the Paleozoic Era (Oji, 2001, 2015), suggesting that this ability was already present in the common ancestor and was a successful strategy throughout their evolutionary history.

Despite their relevance, echinoderms are still far from being routinely used as model systems to investigate regeneration. However, in the last decade an increasing number of molecular tools and data have become available (Ben Khadra et al., 2018b), promoting the profitable use of these animals among regeneration researchers. In the following paragraph, we will review current knowledge on the cell types recruited for regeneration, focusing on adult regeneration of all echinoderm clades. It must be stressed that no cell tracking experiment has ever been conducted in studies of echinoderm regeneration, and most data derive from microscopy (light and transmission electron microscopy) or molecular (e.g., *in situ* hybridization or transcriptomic) analyses. Therefore, what is known about echinoderm regeneration represents “static” snapshots of a continuous process and can hardly provide unequivocal evidence of the origin and fate of the cells involved. Nevertheless, the increasing quantity of data available for these systems is providing some important clues about the processes underlying stem cell-based organogenesis.

### Crinoidea

In the most basal of the echinoderms, regeneration of whole body-parts, i.e., arms and the visceral mass (Figure 2), has been investigated from histological, ultrastructural and molecular perspectives in a few comatulid species (Candia Carnevali and Bonasoro, 2001; Patruno et al., 2003; Mozzi et al., 2006; Kondo and Akasaka, 2010; Shibata et al., 2010; Kalacheva et al., 2017). These approaches allowed the identification of several cytotypes, proteins and genes involved in regeneration. Sea lilies (stalked crinoids) have also exceptional regenerative potential (Nakano et al., 2004), but limited information is available at the cellular level, and they will therefore not be discussed further here.

During arm regeneration in *Antedon mediterranea*, morphologically undifferentiated cells present in the stump tissues (i.e., brachial nerve cortex and coelomic cavities; Figure 2) are recruited to the area where the regenerative blastema will eventually form (Candia Carnevali and Bonasoro, 2001). These include undifferentiated amebocytes, which are satellite elements physiologically present around the brachial nerve, and undifferentiated coelomocytes, a sub-population of circulating cells in the coelomic fluid, likely produced by dedifferentiation of the coelomic epithelia. Both these cell types display a typical undifferentiated phenotype, with a high nuclear/cytoplasmic ratio and mainly euchromatic nuclei, and undergo proliferation (Candia Carnevali et al., 1995, 1997). They differ mainly in their general morphology: amebocytes are rather elongated, apparently migrating, cells, whereas coelomocytes display a more roundish morphology and vesicles. Whether this is simply the result of a different tissue localization or a true cytological difference is currently unknown. These cells are considered presumptive pluripotent stem cells (amebocytes) or progenitor cells (coelomocytes) which, upon trauma, migrate toward the amputation area where they proliferate extensively, thereby contributing to the formation of the blastema. Candia Carnevali and Bonasoro (2001) hypothesized that the undifferentiated coelomocytes are lineage-restricted, giving rise to all the cells associated with the coelomic epithelium (peritoneocytes, myoepithelial cells), whereas the undifferentiated amebocytes have a wider “stemness” potential, generating all the remaining structures. However, the possibility that the blastema cells include several different subpopulations of already committed cells, as described in the case of the urodele limb (Stocum, 2019), cannot be excluded.

Besides the recruitment of undifferentiated cells, dedifferentiation phenomena can also occur during arm regeneration, especially at the level of the muscle bundles (Figure 2). This is rarely observed during arm regeneration under physiological conditions (Shibata et al., 2010); however, it occurs consistently under stress, such as the presence of contaminants, after basal or non-autotomic amputations, in arm explants, etc. (Candia Carnevali and Bonasoro, 2001; Sugni et al., 2007; Di Benedetto et al., 2014b).

During visceral regeneration, transdifferentiation and dedifferentiation of specialized adult cells are the main mechanisms of cell recruitment, but the cells involved differ in the species studied so far. While transdifferentiation of coelomic epithelial cells apparently produces enterocytes in *A. mediterranea* (Mozzi et al., 2006), in *Himerometra robustipinna* the latter are generated by neurosecretory-like cells (juxta-ligamental cells; Kalacheva et al., 2017). In *H. robustipinna*, the employment of remodeling and dedifferentiation of adult cells is further demonstrated by the fact that regeneration normally proceeds even when proliferation is pharmacologically inhibited (Kalacheva et al., 2017). While microscopy-based investigations on the cellular source have been performed in this echinoderm clade, at present no studies have been published on the molecular signature of these cells or the presence and/or expression of classic “stemness” markers. The only available molecular investigation carried out in crinoids suggested the expression of the BMP-like growth factor *anbmp2/4* in *Antedon bifida* regenerating arms (Patruno et al., 2003). Although the true homology of *anbmp2/4* awaits more in depth phylogenetic analyses, these data support a possible involvement of the TGFβ superfamily in cell migration (Patruno et al., 2001), in agreement with its key role during epithelial-mesenchymal interactions in different regenerating animals (Ferretti and Géraudie, 1998).

In general, despite being phylogenetically relevant models and to have exceptional regenerative abilities, there is a remarkable lack of knowledge about crinoids, and they are by far the least studied echinoderm clade, particularly from a molecular perspective. Future studies should aim to address this important gap.

### Echinoidea

Regeneration studies in this clade have mainly focused on pedicellariae, spines, tests (Hobson, 1930; Dubois and Ameye, 2001; Bonasoro et al., 2004) and tube feet (Reinardy et al., 2015; Figure 3). Although differences in terms of numbers and final differentiation were observed depending on the pedicellaria type, regeneration apparently occurs through recruitment of undifferentiated cells (Dubois and Ameye, 2001). In the case of spines, a distinction between basally removed and broken spines should be made (Dubois and Ameye, 2001). In the former case, morphologically undifferentiated cells – regarded as presumptive ASCs – are involved, whereas regeneration of broken spines mainly relies on rearrangement of the stump tissues and dedifferentiation. These same processes are also employed during regeneration of the test, i.e., the calcareous dermaskeleton enveloping most sea urchin organs (Bonasoro et al., 2004). In particular, undifferentiated coelomocytes and amebocytes, as well as differentiated phagocytes, are recruited to the damaged area, and a blastema of undifferentiated, proliferating cells is visible until the complete differentiation of all the missing tissues. A contribution from dedifferentiated myocytes has also been hypothesized (Bonasoro et al., 2004).

Overall, stem cell markers are poorly studied in adult tissue regeneration in this clade. Nevertheless, a recent study on spine and tube foot regeneration of different sea urchin species has shown that *vasa* and *piwi* are present in both structures, suggesting the presence of multipotent progenitor cells in these somatic tissues (Reinardy et al., 2015; Bodnar and Coffman, 2016). Moreover, the *Notch* signaling pathway is essential for both tube foot and spine regenerative processes (Reinardy et al., 2015).

### Holothuroidea

Radial nerve cords and gut are the main tissues studied in sea cucumber regeneration (Gibson and Burke, 1983; García-Arrarás et al., 1998; Mashanov et al., 2008, 2013, 2014; Mashanov V. et al., 2017; Mashanov V. V. et al., 2017; Okada and Kondo, 2019; Figure 4). Regeneration of both structures apparently relies mainly on dedifferentiation and subsequent re-differentiation processes. In the radial nerve cords, the supporting cells (radial glial cells) close to the amputation site react to injury by dedifferentiating and then re-differentiating into the same cytotype, as well as into newly specialized neurons (Mashanov et al., 2008, 2013). In this sense, the radial glial cells can be considered a differentiated local source of new neural elements as well as new supporting cells necessary for the regrowth of the nerve structure (Mashanov and Zueva, 2019). As such, their potency would be rather restricted. Besides local radial glial cells, a contribution of migrating cells from more “distant” regions of the stump is also present, although their nature remains to be clarified (Mashanov V. et al., 2017). Indeed, radial nerve cord regeneration occurs even after proliferation is inhibited, thanks to cell recruitment from stump tissues, suggesting that the balance between cell migration and proliferation is highly plastic and finely regulated, eventually ensuring the complete restoration of the missing structures. The absence of “stemness” transcripts during radial nerve cord regeneration further supports the major employment of reprogramed adult differentiated cells rather than the recruitment of resident adult undifferentiated cells (Mashanov et al., 2014).

During gut regeneration, dedifferentiation mainly occurs in muscle tissue (Candelaria et al., 2006; García-Arrarás and Dolmatov, 2010) and cell supply is ensured through epithelial-mesenchymal transition (EMT; see glossary) (García-Arrarás et al., 2011). Mesothelial cells ingress in the underlying connective tissue layer and become mesenchymal cells that then migrate toward the regenerating intestine. Regeneration of missing parts (e.g., neural cord/ring, digestive tract, water vascular system) after fission in *Cladolabes schmeltzii* occurs *via* dedifferentiation, proliferation and migration of the respective remaining ends (Kamenev and Dolmatov, 2017). Here, epithelial morphogenesis is the key regenerative mechanisms that allows reconstruction of the missing body parts, and regeneration is basically restricted within cell/tissue types.

Dedifferentiation is also evident from molecular analyses with the use of specific markers identified in the regenerating transcriptome of *Apostichopus japonicus* (Sun et al., 2011). Genes and proteins linked to cell migration, proliferation and differentiation have been detected in *Holothuria glaberrima* intestinal regeneration during the first 2 weeks of regeneration (Rojas-Cartagena et al., 2007; Ortiz-Pineda et al., 2009; Mashanov et al., 2012). Mashanov et al. (2015) observed the expression of pluripotency factors/markers in adult uninjured tissues of the sea cucumber *H. glaberrima* as well as in regenerating tissues, although a specific coordinated regulation is not evident. In particular, *soxB1* is downregulated during gut regeneration, whereas *myc* is upregulated in both regenerating gut and radial nerve cord, suggesting that dedifferentiation of adult cells occurs in both tissues but depends on different gene regulatory pathways (Mashanov et al., 2015). Furthermore, homologs of mammalian intestinal stem cell markers such as *Bmi1* are apparently expressed in both luminal epithelium and mesothelium (coelomic epithelium) of non-regenerating digestive tube, in particular in the peritoneocytes of the coelomic epithelium (Mashanov V. V. et al., 2017). Besides putative pluripotency factors, Li et al. (2017) studied the dynamic expression changes of *Wnt* signaling pathway ligand WntA during *A. japonicus* intestinal regeneration. The correlation between *WntA* expression and cell cycle activity at different stages led the authors to suggest that this gene might participate in wound healing and regeneration, possibly *via* either direct or indirect influences on cell proliferation and apoptosis.

### Ophiuroidea

Regeneration of autotomized and traumatically amputated arms as well as arm explants has been extensively studied in this clade starting in the early 1900s (Dawydoff, 1901; Zeleny, 1903; Morgulis, 1909; Thorndyke et al., 2003; Dupont and Thorndyke, 2006; Biressi et al., 2010; Duque-Alarcon, 2015; Czarkwiani et al., 2016; Ferrario et al., 2018; Figure 5). Recent studies have shown that a true blastema of mesenchymal and scattered undifferentiated cells is not present (reviewed in Ben Khadra et al., 2018b). Rather, the regenerative bud is mainly formed by the outgrowth of the main axial structures (aboral coelomic cavity, water vascular system and radial nerve cord), whose cells undergo dedifferentiation and acquire an undifferentiated morphology, although they maintain their epithelial features (Biressi et al., 2010; Czarkwiani et al., 2016). Once dedifferentiated, after the end of the repair phase, these cells start to proliferate, as demonstrated by 5-bromo-2’-deoxyuridine (BrdU) and 5-ethynyl-2’deoxyuridine (EdU) labeling experiments. Proliferating cells are always present at the tip of the regenerate, just behind the differentiated terminal ossicle, suggesting that the distal-most tips of the three axial structures are actively involved in the constant re-growth of the structures themselves and of the regenerates (Biressi et al., 2010; Czarkwiani et al., 2016; Canavesi, 2018). Therefore, unlike crinoids and similarly to holothuroids, echinoids and asteroids (see below), regeneration mainly relies on recruitment of adult differentiated cells *via* dedifferentiation. It has been suggested that cells generating sclerocytes are recruited from the aboral coelomic cavity epithelium, migrate as progenitor-like cells and re-differentiate *in situ* (Piovani, 2015). In this case, EMT may occur to ensure the recruitment of new cells.

Muscles are largely used as a source of putative dedifferentiating myocytes (Biressi et al., 2010; Czarkwiani et al., 2016). Muscle remodeling has also been detected molecularly in *A. filiformis*, where a zonadhesin-like protein has been identified, particularly in the first stages of arm regeneration (Burns et al., 2011; Purushothaman et al., 2015). In the same species, two genes involved in cell migration are expressed in cells within the radial water canal of the regenerate, suggesting the importance of the radial water canal as a source of cells for regeneration (Bannister et al., 2005, 2008). However, histological and ultrastructural observations suggest that the aboral coelomic cavity epithelium is the main provider of the cellular material involved in regeneration (Biressi et al., 2010; Piovani, 2015; Czarkwiani et al., 2016). Regardless of their origin, cells of the regenerate require the proper orchestration of several processes, including cell migration and proliferation, as well as an appropriate extracellular matrix environment and immune system signals (Ferrario et al., 2018, 2020). Mashanov et al. (2020) recently proposed the *Notch* pathway as a putative key director of this signaling cross-talk. Further analyses will be crucial to improve our understanding of the origin of cells involved in brittle star regeneration.

### Asteroidea

Arm explant and arm regeneration, after both traumatic and auto-induced mutilations, have been investigated to understand which cells, genes and proteins are involved in these processes (Figure 6). Recruitment of adult resident undifferentiated cells is much less evident in asteroids than in crinoids: the pyloric caeca and the coelomic epithelium have been proposed as sources of presumptive stem/progenitor cells, but in both cases dedifferentiation of the highly specialized cells of these tissues probably occurs prior to recruitment (Hernroth et al., 2010; Sharlaimova et al., 2010; Sharlaimova and Petukhova, 2012; Ben Khadra et al., 2015b, 2017, 2018b). As described for the ophiuroids, at the onset of the early regenerative phase, the dedifferentiated cells at the tip of the re-growing structures, as well as epidermal cells, undergo intense proliferation (Mladenov et al., 1989; Moss et al., 1998). Stump tissue rearrangement and cell dedifferentiation are much more commonly employed, especially in the case of muscle tissues (Ben Khadra et al., 2015a,b, 2017). Therefore, the coelomic myoepithelia might be regarded as one of the cellular sources for arm regeneration, while the free wandering undifferentiated coelomocytes may be tissue-specific stem cells producing only other coelomocytes (Sharlaimova et al., 2014; Ben Khadra et al., 2018b). Cells recruited from these tissues perform EMT to actively migrate within the dermal tissue toward the regenerating area, possibly recruited by specific signals coming from the damaged region (Ben Khadra et al., 2018b).

The few data available on “stemness” markers are not related to adult regeneration but to that of the bipinnaria larvae of *Patiria miniata*, where a *vasa* gene has been identified (Oulhen et al., 2016). Recent work has also shown that genes involved in a diverse array of pathways are expressed during anterior and/or posterior larval regeneration at different stages (Cary et al., 2019), suggesting that molecular signaling commonalities might exist between sea star larval regeneration and whole body regeneration of other metazoans.

In addition to “stemness” markers, the expression of *Wnt* genes have been detected during *Echinaster sepositus* arm regeneration, in particular during the first 3 days after damage and late during arm re-growth (Ben Khadra et al., 2018a), suggesting their involvement during both wound healing and morphogenetic processes. Ferrario et al. (2018) also isolated a fibrinogen-like gene in this species, underscoring the importance of the immune system in the initial phases of regeneration.

## Hemichordata

**FIGURE 7:**
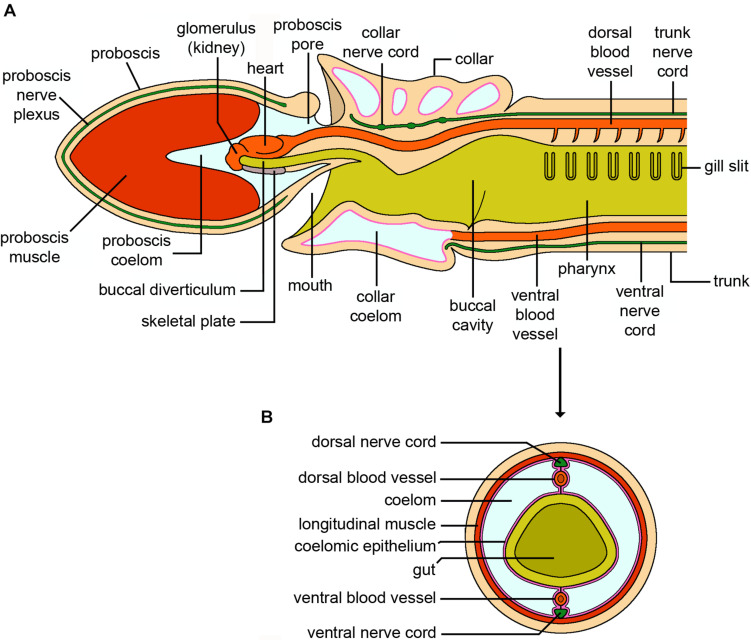
Hemichordata. **(A)** Schematic longitudinal section of an adult solitary enteropneust hemichordate (*Ptychodera*). Only the internal anatomy of the proboscis (prosome), collar (mesosome) and the anterior part of the trunk (metasome) containing the branchial region are shown. The external gill pores, genital wings with gonads, hepatic sacs and posterior trunk with terminal anus have been omitted for clarity. **(B)** Cross section through the body wall posterior to the branchial region. The dorsal and ventral nerve cords and associated blood vessels are easily distinguished (credits: Alessandro Allievi).

Unlike echinoderms, from which they diverged 559 Mya (Simakov et al., 2015), hemichordates have a more archetypical body plan with clear bilateral symmetry and anteroposterior identity (Figure 7). Within the phylum, the two clades Enteropneusta (acorn worms) and Pterobranchia show a diversity of lifestyles, with solitary and tubiculous colonial forms, respectively (Röttinger and Lowe, 2012). Recent fossil evidence of a stem echinoderm, *Yanjiahella biscarpa*, suggests that the enteropneust body plan is ancestral within the hemichordates (Topper et al., 2019), indicating that enteropneusts might be most informative for highlighting any conserved mechanisms across ambulacrarians. Although there is currently no information about regeneration in pterobranchs (Rychel and Swalla, 2009), their asexual mode of reproduction by budding and colony regeneration after episodes of mortality (Rigby, 1994) suggest that they are likely to regenerate well, as do many colonial tunicates (see below). This is supported by extensive fossil data of regeneration in the extinct graptolites (e.g., Urbanek, 1963, and many others), now considered to be related to modern rhabdopleuran pterobranchs (Mitchell et al., 2013). In contrast, regenerative ability is well documented and widespread in adult enteropneusts, particularly in the indirect developing Ptychoderidae (e.g., Willey, 1899; Dawydoff, 1909, 1948; Rao, 1955; and reviewed extensively in Rychel and Swalla, 2009). The direct developing harrimaniid enteropneusts, on the other hand, appear to regenerate less well than ptychoderids (Tweedell, 1961) or not at all (Rychel and Swalla, 2009). To our knowledge, there are no data on regeneration in the Torquaratoridae, but in the Spengelidae *Glandiceps hacksi* is reported to autotomize and regenerate the caudal portion (Urata et al., 2012). Evidence of asexual reproduction by fission and paratomy in different groups likely goes hand in hand with regenerative ability (Miyamoto and Saito, 2010; Worsaae et al., 2012). Here, we will describe the current state of the art of regeneration research on enteropneusts, and where known, the cellular and molecular players in the process.

Regeneration of anterior structures is generally considered to be more common than posterior regeneration in hemichordates (Rychel and Swalla, 2009). However, regenerate success and quality depend on the level of amputation or autotomy, the system studied, and the health of individuals (Willey, 1899; Tweedell, 1961; Nishikawa, 1977; Rychel and Swalla, 2009; Humphreys et al., 2010; Urata et al., 2012; Arimoto and Tagawa, 2018). As in other systems, regenerative success may also vary according to animal maturity (e.g., Tweedell, 1961) or developmental stage, as tornarian larvae of *Ptychodera flava* can regenerate when cut along the axial, sagittal and coronal planes (Luttrell et al., 2018). In most cases, both proliferation-dependent processes and tissue remodeling are assumed, but not always clearly demonstrated. For instance, blastemas have been described during anterior regeneration in *Balanoglossus simoidensis* (Miyamoto and Saito, 2010), but proliferation has only been carefully analyzed in *P. flava*, where dividing cells have been clearly labeled with PCNA antibody in the epidermis and mesenchyme of the trunk “coelom” during proboscis and collar regeneration (Rychel and Swalla, 2008). After proboscis regeneration, an “insertional blastema” appears between the new proboscis and the mature body (Humphreys et al., 2010). Gill slits form in areas previously shown to be hepatic sacs, with increased apoptosis of endoderm as assayed by TUNEL (Rychel and Swalla, 2008), suggestive of tissue remodeling. Mobilization of stem cells at a distance from the wound site also cannot be ruled out.

In hemichordates, there is so far no evidence of neoblast-like or totipotent stem cells possessing the characteristically large nuclear/cytoplasmic ratios. During regeneration of the proboscis in adult *B. simoidensis* (Miyamoto and Saito, 2010), the blastema is filled with apparently undifferentiated cells. Any fragments containing genital or branchial regions (and which include gonads) regenerate completely with rapid wound healing and blastema formation. In contrast, animals that lack such fragments – although they can survive for long periods – show delayed wound healing and blastema formation processes and are generally unable to form lost body parts. Few mesenchymal cells were seen associated with the cut surfaces in this case. Mesenchymal-like (undifferentiated) cells appear throughout the trunk (Miyamoto and Saito, 2010) and also contribute to regenerating structures in *P. flava* associated with the nerve layer (Rychel and Swalla, 2008), but their origins are unclear. Evidence that regeneration occurs in fragments with gonads may also suggest migration and contribution of germ-like cells, although neither hypothesis has been formally tested. Citing unpublished EST and gene expression data, Arimoto and Tagawa (2018) argue that hemichordate regeneration is likely dependent upon dedifferentiated cells reacquiring multi/pluripotency, rather than the existence of resident stem cells. So far, there is no conclusive evidence for direct transdifferentiation from one cell type to another in hemichordates. However, while posterior regeneration by amputation of the trunk through the hepatic region (which removes the pygochord) in *P. flava* does not produce an obvious blastema, the pygochord nevertheless regenerates. The pygochord is a vacuolated chord-like midline structure, associated with the ventral wall of the hindgut, and located within the pre-anal posterior region of some enteropneusts (Willey, 1899). Its evolutionary origin and homology are still unclear (Willey, 1899; Annona et al., 2015; Yoshimura et al., 2019), but elucidating the cellular origins of the regenerating pygochord may help shed light on these problems. During regeneration, it arises quite late in the process [14 days post-amputation (dpa)] ventrally from the gut wall, associated closely with a blood vessel between the gut epithelium and the ventral nerve cord. This, combined with gene expression (see below) and the loss of the hepatic sacs during regeneration may support transdifferentiation (Yoshimura et al., 2019). Alternatively, it might suggest the existence of circulating stem cells associated with the blood vessel, similar to the hemoblasts seen in tunicates such as *Botryllus schlosseri* (Ballarin and Cima, 2005).

The few molecular data that exist for hemichordate regeneration have been generated in *P. flava*. Luttrell et al. (2016) amputated adults between the genital wings and the hepatic sacs to study gene expression profiles during the first 4 days of anterior regeneration. They uncovered complex patterns of differentially expressed gene clusters, a large percentage of which play roles in differentiation, cell proliferation and morphogenesis, or are part of Wnt, FGF and Notch signaling pathways. So far, none of these putative players has been validated *in situ*. However, Arimoto and Tagawa (2018) report ongoing expression studies of some of the gene families related to vertebrate pluripotency factors (such as *Klf, Sox* and POU domain transcription factors) that were previously identified as differentially expressed (Luttrell et al., 2016). In such a candidate approach, Humphreys et al. (2010) that *SoxB1* is expressed in the nascent proboscis. Similarly, *Hedgehog* (*Hh*) is expressed in the pharyngeal region, reminiscent of its expression during development (Arimoto and Tagawa, 2015). However, the absence of *Hh* expression in the anterior tip of the regenerating proboscis during regeneration was unexpected, leading the authors to suggest that in enteropneusts, Hh signaling plays a role specific to the regeneration process (Arimoto and Tagawa, 2015). We were unable to identify any members of the Hh pathway in the up- or down-regulated gene clusters reported in the large-scale transcriptional profiling study of Luttrell et al. (2016). Although this does not exclude the possibility that this reflects limitations of study design or statistical power, the data lend support to the idea that anterior regeneration does not strictly recapitulate the developmental program in *P. flava* (Luttrell et al., 2016). This may also reflect a general lability in the timing of regenerative events both within the species and relative to development, specifically when comparing the sequence of appearance of the nerve cord, the collar, the proboscis and the gill slits (Nielsen and Hay-Schmidt, 2007; Humphreys et al., 2010; Luttrell et al., 2016). In any event, the identification of differentially expressed transcription factors associated with brain formation in chordates, including homeobox factors, paves the way for further study comparing anterior regeneration and development in hemichordates. Finally, the regenerating pygochord expresses a unique combination of genes distinguishing it as having a specific cellular identity (*Fcol^+^, MHC^–^, elav^+^*) relative to muscle (*Fcol^+^, MHC^+^*), or gut epithelium (*Fcol^–^, MHC^–^*), but shared with some gut cells and the ventral nerve cord (*elav*^+^; Yoshimura et al., 2019). It is not clear if some of these *elav*^+^ gut cells are in fact neurons embedded within the gut epithelium, but this intriguing result may suggest that the pygochord dedifferentiates from the gut epithelium (Yoshimura et al., 2019). Additional molecular markers might help resolve the origins of the regenerating pygochord.

## Cephalochordata

**FIGURE 8:**
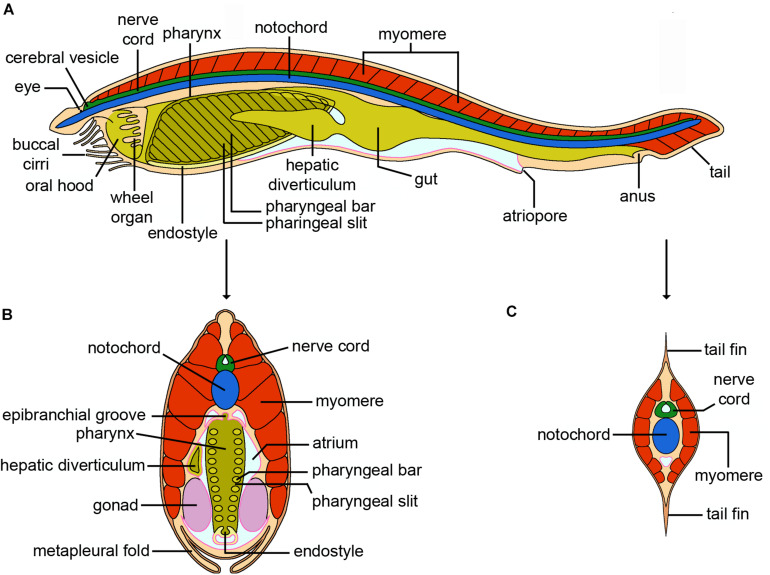
Cephalochordata. **(A)** Schematic drawing of an adult cephalochordate (*Branchiostoma*). The gonads and lateral musculature (myomeres) overlying the nerve cord, notochord and digestive system have been omitted for clarity. Unlike other chordates, the notochord is a muscular structure in cephalochordates. The thickening of the anterior nerve cord corresponding to the brain is called the cerebral vesicle, and has a single photoreceptor at its terminus, the frontal eye. The circulatory system is not shown. **(B)** Cross section through the pharyngeal region. Although generally considered to be bilaterally symmetric as adults, with rows of gonads running along either side of the body, the hepatic diverticulum is located on the left side of the pharynx/branchial basket. The endostyle, which runs along the base of the pharynx, is a homolog of the vertebrate thyroid. **(C)** Cross section through the post-anal tail. Note the dorsal and ventral tail fins and the proportionally greater size of the nerve cord and notochord (credits: Alessandro Allievi).

Cephalochordates (Clade Leptocardii; also called “amphioxus” or “lancelets”) are the earliest diverging invertebrate chordates (Figure 1A) and share the most similar body plan to that of vertebrates (Bourlat et al., 2006; Delsuc et al., 2006, 2008; Figure 1B). The three extant genera of cephalochordate (*Asymmetron*, *Branchiostoma* and *Epigonichthys*) include 30 or so species, all of which are considered to belong to a single family, the Branchiostomatidae (Poss and Boschung, 1996). Regeneration has been described in a number of species of *Branchiostoma* as well as in *Asymmetron lucayanum* (Andrews, 1893; Probst, 1930; reviewed in Somorjai, 2017), most notably of the tail, a key chordate feature consisting of notochord, dorsal nerve cord and segmented musculature (Figure 8). Anterior regeneration, or posterior regeneration of animals amputated anterior to the anus, are generally poor (Somorjai et al., 2012b).

Tail regeneration in *Branchiostoma lanceolatum* and *B. japonicum* is considered to occur *via* the formation of a true blastema (Somorjai et al., 2012b; Liang et al., 2019) consisting of at least superficially undifferentiated proliferating cells. Treatment with hydroxyurea, an inhibitor of DNA replication, in the early stages of regeneration (2-5 dpa) results in smaller tails, further supporting a role for cell proliferation (Wang et al., 2019). The source is still unknown, but may include a population of resident stem cells associated with myofibres, termed muscle satellite-like stem cells, dedifferentiated muscle fibers generated as the myosepta near the amputation plane degenerate (Somorjai et al., 2012b), or even coelomocytes. In contrast, the regenerating nerve cord may arise from proliferating nerve cord precursors directly, as described for some echinoderm species. The notochord – a muscular rod in amphioxus that is maintained into adulthood – appears to employ a dedifferentiation process, as the differentiated “stack of coins” appearance typical of the mature notochord is lost anterior to the amputation plane in the early stages, reappearing later as the regenerating notochord elongates and differentiates (Somorjai et al., 2012b). However, the contribution of notochord stem cells or progenitors cannot be ruled out. In any case, faithful regeneration can be induced multiple times in the same animal (Somorjai et al., 2012a), indicating that any stem cell reservoirs involved are not limiting, at least in young animals. Genetic lineage tracing will be required to really identify the different cellular mechanisms underlying this complex regeneration process.

In contrast to the tail, the regeneration of the oral cirri (Kaneto and Wada, 2011; Somorjai et al., 2012a,b) – non-mineralized skeletal rods surrounding the mouth opening – may proceed without blastema formation, as no increase in cell proliferation was observed in regenerates compared to uncut cirri using an antibody for phosphorylated Histone H3, a marker for cells in the M-phase (Kaneto and Wada, 2011). In this case, the mesenchymal cells contributing to the regenerating cirri must arise from alternative cellular sources at a distance from the wound *via* migration. Alternatively, slow cycling stem cells may simply not have been labeled by the methodology employed.

The molecular basis of regeneration in cephalochordates is still poorly characterized, but transcriptomic data in *B. lanceolatum* and *B. japonicum* indicate that signaling pathways such as BMP, Wnt and Notch are involved (Dailey, 2017; Somorjai, 2017; Liang et al., 2019), as well as ROS (Dailey, 2017; Liang et al., 2019), an important conserved early signal in a number of regeneration contexts linking apoptosis and proliferation to wound healing and regeneration (Pirotte et al., 2015; Romero et al., 2018). Of these, Wnt and BMP are the best characterized. Broad expression of *wnt5* and accumulation of beta-catenin protein in the membranes of the tail blastema cells may argue for a role of non-canonical Wnt signaling in regeneration (Somorjai et al., 2012a). Conversely, identification in the blastema of transcripts of *sp5*, a downstream target of beta-catenin-dependent Wnt signaling during amphioxus development, suggests that canonical Wnt function also operates during regeneration (Dailey et al., 2017). *Msx*, a marker for undifferentiated cells as well as a target of BMP signaling, and *chordin*, a BMP antagonist, are also expressed in *B. lanceolatum* regenerates (Somorjai et al., 2012b). Recently, it has also been shown that *bmp2/4* is expressed in wounds in *B. japonicum*, both those that induce regeneration and those that do not, suggesting a more general role in the repair process and not just regeneration *per se* (Liang et al., 2019). In this context, results showing that the implantation of Noggin-soaked beads at the amputation site and injection of *bmp2/4* morpholinos – both of which should reduce BMP signaling – cause degeneration of tails (Liang et al., 2019) deserve further attention. Other genes expressed during tail regeneration include *soxB2*, the cephalochordate ortholog of *sox17/21* in vertebrates, and *pax3/7* (transcripts and protein). Both are expressed in the nerve cord, while *pax3/7* is also expressed in blastema cells and in cells that might constitute muscle satellite-like stem cells (Somorjai et al., 2012b). There are in fact two Pax3/7 genes in amphioxus, *pax3/7a* and *pax3/7b*, arising from a cephalochordate-specific tandem duplication event, and which were originally identified in a tail regenerate transcriptome in *B. lanceolatum* (Somorjai, 2017; Barton-Owen et al., 2018). Studies elucidating their differential roles during regeneration are currently underway.

Cirrus regeneration is much less well characterized than tail regeneration molecularly. Skeletogenesis genes *soxE* and *runx*, as well as extracellular matrix (ECM) genes including *SPARC/SPARCL* and the fibrillar collagens *fcol1* and *fcol2*, are expressed in mesenchyme cells during oral cirrus regeneration in *B. japonicum* (formerly classed as *B. belcheri*) (Kaneto and Wada, 2011), suggesting a recapitulation of developmental gene programs, similarly to tail regeneration. However, how the molecular and cellular processes underlying regeneration in amphioxus are integrated remain unknown. Detailed analyses of the expression patterns of more genes identified using transcriptomic approaches during regeneration will be invaluable in our understanding of the cellular basis of regeneration in cephalochordates.

## Tunicata

Tunicates or urochordates are invertebrate chordates considered the sister group of vertebrates (Bourlat et al., 2006; Delsuc et al., 2006, 2008; Figure 1A). They are marine filter-feeders, benthic or pelagic, classically subdivided into Ascidiacea (ascidians), Thaliacea (salps and pyrosomes) and Larvacea (appendicularians), although the internal interrelationships among the various taxa are still controversial (Stach et al., 2010). Tunicates owe their name to the distinctive covering embedding the body -the tunic- a cellulose-containing structure unique in the animal kingdom (Deck et al., 1967; Welsch, 1984; Van Daele et al., 1992), whereas the name “urochordates” comes from the notochord, the supporting rod characterizing chordates, here limited to the larval muscular tail. Almost all tunicate species have a swimming tadpole-like larva that metamorphoses into a highly derived and specialized juvenile, with a dramatic change of body organization (Stolfi and Brown, 2015).

Tunicates include both solitary and colonial species (Figure 1B): the latter are unique among chordates as they are capable of asexual reproduction by budding (Brown and Swalla, 2012). Their particular phylogenetic position has attracted considerable interest; however, the regenerative capabilities of the group have only been studied in a handful of species of solitary and colonial ascidians. Regeneration studies started in the late XIX century as investigators/scientists were fascinated by the ability of ascidians -unusual among metazoans- to regenerate a functional brain (Berrill, 1951; Jeffery, 2015a). Today, the availability of genomes and transcriptomes of an increasing number of tunicate species is leading to new analyses of the regenerative process and a better understanding of the molecules and signaling pathways involved. Below, we provide an updated review of the main advances in our knowledge of regeneration in ascidians.

### Solitary Ascidians

#### Tunic Regeneration

The tunic can easily be detached from the body wall. Old experiments demonstrate that, at least in *Ciona intestinalis*, *Ascidia mentula* and *Ascidiella aspersa*, it is easily and rapidly reformed by the underlying epidermis (Fol, 1908; Azéma, 1927; Pérès, 1948).

#### Partial Body Regeneration

**FIGURE 9:**
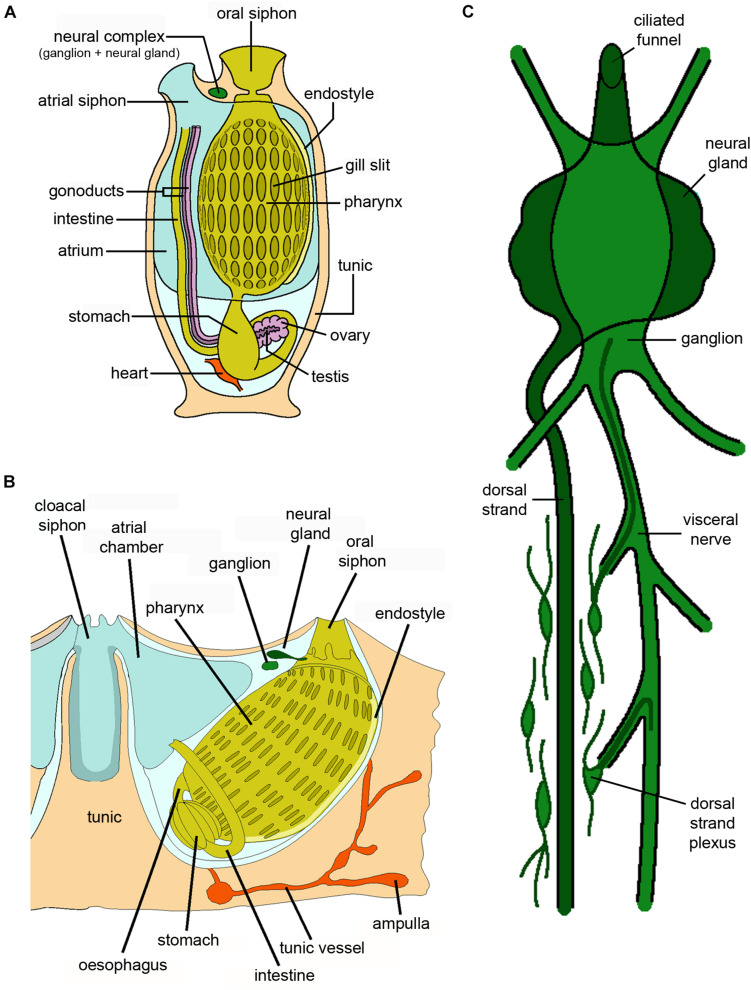
Schematic drawings showing the anatomy of **(A)** a solitary ascidian, **(B)** a botryllid colonial ascidian, and **(C)** the organization of the neural complex of a solitary ascidian (credits: Alessandro Allievi).

Solitary ascidians (Figures 1B, 9A) are capable of partial body regeneration (Gordon et al., 2019). Jeffery and collaborators have studied the process in detail in adults of the species *Ciona robusta*, previously referred as *Ciona intestinalis* type A (Caputi et al., 2007). When animals are bisected, the posterior (proximal) region of the body, containing viscera, can regenerate the anterior (distal) part, including the brain, provided that it contains at least a part of the pharynx. Conversely, the anterior part of the body cannot regenerate any of the proximal structures (Jeffery, 2015a,b). Even when the animal is cut in three parts along the proximo-distal axis, the middle section can reform the distal part (Jeffery, 2015b). This implies that the pharynx is important for regeneration, and is crucial for the replacement of distal body parts.

Regeneration of the oral siphon in *Ciona* received considerable interest in the past (Wermel, 1930; Sutton, 1953; Whittaker, 1975). Recently, Jeffery (2015a, b) demonstrated that both short-distance and long-distance processes are involved in the process. Short-distance regeneration occurs when the siphon is amputated at its tip, and leads to the replacement of the oral pigment organs (OPOs) and of the very distant part of the siphon. This kind of regeneration does not require cell proliferation; neither labeling with the cell proliferation maker EdU nor effects of proliferation inhibitors colchicine or nocodazole are observed (Jeffery, 2015b). It relies on small aggregates of stem/progenitor cells already present in the siphon, activated by the injury (Auger et al., 2010; Jeffery, 2015b).

Long distance regeneration leads to the formation of new circular muscle fibers and neurons, and requires the activity of stem/progenitor cells originating in the pharyngeal region. These migrate distally where they form a blastema, with a well-defined proliferation zone, in the proximal region of the siphon stump (Auger et al., 2010). When the siphon is amputated at its base, only long-distance regeneration occurs, with stem/progenitor cells from the pharyngeal region forming both the blastema and the OPOs (Jeffery, 2015b).

The stem/progenitor cells originate in the lymph nodes, typical stem cell niches located in the transverse vessels of the pharynx, where alkaline phosphatase positive, *piwi*-positive and EdU-labeled cells reside (Jeffery, 2015b). The lymph nodes are hematopoietic organs, involved in the renewal of the circulating hemocytes (Ermak, 1976). From the pharynx vessels, EdU positive-cells migrate into the regeneration blastema after the amputation of the siphon. This has been confirmed by transplanting the pharynx from small animals, labeled with EdU, into the pharynx of larger animals: in this case EdU-labeled cells can be found in the regeneration blastema (Jeffery, 2015b). Regenerative abilities decline with age, up to their complete disappearance, due to the depletion of stem cells in the branchial sac, as supported by the severe reduction of alkaline phosphatase- and *piwi*-positive cells in the pharyngeal region (Jeffery, 2015c,d).

Regenerative activity requires the *Notch* signaling pathway: specific inhibitors can inhibit stem cell proliferation and muscle differentiation (Hamada et al., 2015). In addition, the *TGF*β signaling pathway is also required, as pathway-specific inhibitors completely block regeneration (Spina et al., 2017). Moreover, during regeneration, a number of miRNAs involved in the modulation of *Wnt*, *TGF*β and *MAPK* signaling are expressed (Spina et al., 2017). The underlying epidermis forms the new tunic (Jeffery, 2015a).

As reported above, an outstanding feature of solitary ascidians is their ability to regenerate the central nervous system, which in adult animals is formed by the cerebral ganglion, lying above the front end of the pharynx between the two siphons. It is usually associated with the neural gland, which opens on the roof of the pharynx with its ciliated duct and the dorsal strand, an epithelial organ located at the caudal-most part of the gland (Burighel and Cloney, 1997; Figure 9C). Collectively, the cerebral ganglion and the neural gland form the neural complex (NC), which undergoes complete regeneration within a month after its ablation (Dahlberg et al., 2009). Four stages have been identified in the regeneration of the neural complex: i) wound healing, ii) merging and growing of nerves toward the wound region, iii) structural regeneration of the ganglion and iv) functional regeneration and recovery of all the neural complex structures. The growth of nerves is associated with the gathering and proliferation of stem/precursor cells at the tips of the ablated nerves. The origin of these cells is still a matter of debate: they may be undifferentiated hemocytes leaving the circulatory system, undifferentiated cells migrating from the dorsal strand, where extensive proliferation has been observed upon NC ablation, or cells recruited from the mini-ganglia along the nerves, outside the ganglion (Dahlberg et al., 2009).

Even the gonads can regenerate in *Ciona*, implying that germ cells derive from somatic stem cells located outside the gonad that can regain pluripotency (Bourchard-Madrelle, 1966; Jeffery, 2015a). This assumption was recently confirmed by the observation that somatic cells can be converted into germ cells by the removal of primordial germ cells at the larval stage, by cutting off the portion of the tail in which they reside (Yoshida et al., 2017). Complete regeneration of the siphons has also been observed in *Polycarpa mytiligera*, *Styela plicata* and *Herdmania momus* (Gordon et al., 2019).

An unusual type of regeneration has been observed in the species *Polycarpa tenera* and *P. mityligera*, which can eject their viscera as a defense mechanism when subjected to stress conditions. *P. mityligera* can rebuild the branchial sac and gut in less than 20 days (Shenkar and Gordon, 2015), but studies on the cells and molecules involved in the process are still lacking.

### Colonial Ascidians

Colonial ascidians (Figures 1B, 9B), together with pyrosomid Thaliaceans, are the only chordates capable of asexual reproduction. The ability to produce new individuals by various types of budding (reviewed in Kürn et al., 2011; Alié et al., 2020) suggests the presence/recruitment of stem cells or the ability of somatic cells to de-differentiate and re-acquire stem cell properties. Furthermore, in addition to partial body regeneration, colonial ascidians have the capacity for whole body regeneration. Usually, regeneration is not common in compound ascidians, as damaged or injured zooids are simply resorbed and new buds will mature to functionality to replace them. However, in botryllid ascidians, both partial and whole body regeneration have been described.

#### Partial Body Regeneration

##### Zooid regeneration

Several old studies deal with regeneration of zooids after amputation in *Clavelina lepadiformis* and *Archiascidia neapolitana* (Brien, 1930, 1932). In both species, regeneration occurs in both the anterior and posterior cut surfaces and requires the proliferation of cells of the pharyngeal or epicardial epithelium, the epicardium being a thin ventral cavity of pharyngeal origin in the zooid abdomen (Berrill, 1948).

##### Blastogenetic regeneration

In styelid ascidians, palleal budding, i.e., the formation of buds from the lateral mantle (formed by the epidermis, the peribranchial epithelium and the connective tissue between them) is the most common type of budding. In these animals, so-called “blastogenetic regeneration” has been described (Sugino and Nakauchi, 1987). The term indicates the regeneration of a colony from fragments of buds which, after healing of the cut surfaces, emit new buds before being progressively resorbed. The process was initially described in *B. schlosseri* (Majone, 1977). In this species, three blastogenetic generations are usually present in a colony: adult, filtering zooids, their buds stemming from the mantle sides and the budlets on buds (Manni et al., 2007). Colonies undergo cyclical (weekly at 20°C) generation changes during which adults are progressively resorbed and replaced by their buds, which reach adult size and open their siphons; meanwhile, budlets become buds and a new budlet generation appears (Manni et al., 2007). When, in young colonies, both adults and budlets are removed as well as the posterior part of the buds, the anterior bud fragment, -containing the oral siphon, the neural complex, and parts of the branchial basket and the endostyle- can regenerate a whole zooid. It remains connected to the tunic circulation *via* the radial vessel, which regresses within 24 h post-operation, and new vessels sprouted from the marginal vessel connecting the bud fragment. In the subsequent 4 days, the internal tissues lose their morphology and progressively transform into a mass of cells. Five to 6 days after the operation, several new budlets have sprouted from the original bud remain: only one of them gives a distinguishable bud, able to reach adulthood (Majone, 1977). A similar regeneration process has been described in *Symplegma reptans* (Sugino and Nakauchi, 1987) and was also reported in *Polyandrocarpa misakiensis* (Oda and Watanabe, 1982; Sugino and Nakauchi, 1987). No data on the cell types or the genes involved in blastogenetic regeneration are present in the literature. However, recent studies on whole body regeneration (see below) can shed some light on the aforementioned processes.

##### Colonial circulatory system regeneration

The colonial ascidian *B. schlosseri* is able to reform the tunic and the colonial vasculature within 24–48 h of experimental removal (Zaniolo and Trentin, 1987; Gasparini et al., 2008, 2014; Tiozzo et al., 2008). CCS regeneration is preceded by the proliferation of epidermal cells, as revealed by staining with anti-PCNA antibodies, and the formation of new tunic in the damaged region (Gasparini et al., 2008). Both cells detaching from the epidermis and hemocytes entering the tunic contribute to reform the normal tunic cell endowment. Vessel regeneration occurs by sprouting from the vessel remnants and is stimulated by vertebrate vascular endothelial growth factor (VEGF) and epidermal growth factor (EGF) injected into the circulatory system. In addition, antibodies raised against vertebrate fibroblast growth factor-2 (FGF-2), VEGF, EGF and the receptors VEGFR1 VEGFR2 and EGFR recognize the apex of the tubular sprouts (Gasparini et al., 2008, 2014). The involvement of the *VEGF* pathway has been confirmed by the observation that both knock-down of the *Botryllus* VEGF receptor (VEGFR) gene and chemical inhibition of VEGFR block vascular regeneration (Tiozzo et al., 2008). Cell tracing methods suggest that regeneration is supported by the proliferation of vascular resident cells without the contribution of mobile progenitors (Braden et al., 2014).

##### Whole body regeneration

In this type of regeneration, fragments of a colony containing only the colonial matrix (i.e., the tunic and part of the colonial vasculature) can form new buds (and therefore new zooids) from aggregates of circulating cells. These possess characteristic features of stem cells, such as small size and high nucleus/cytoplasm ratio, and are in contact with the epidermis lining the vasculature (Rinkevich et al., 1995, 2007a,b; Voskoboynik et al., 2007; Brown et al., 2009).

One of the first reports of WBR is that of Berrill and Cohen (1936) in *Clavelina lepadiformis*. In this species, experimental fragmentation of the stolon leads to the formation of new zooids, provided that the stolon is of adequate size. Circulating cells of the stolon fragment aggregate and reorganize to form an empty vesicle lined by the stolon epidermis, a situation similar to the double vesicle stage of botryllid ascidians (see below). WBR has also been reported in *Clavelina moluccensis* (Davis, 1988).

In *B. schlosseri*, WBR occurs only after the extirpation of all zooids and buds from the colonial matrix in colonies approaching or undergoing the generation change (Milkman, 1967; Sabbadin et al., 1975; Voskoboynik et al., 2007; Kürn et al., 2011; Ricci et al., 2016). Buds maintain the asymmetry of the parental colony, suggesting a role for the colonial matrix in the transmission of bilateral asymmetry to the newly formed vascular buds (Sabbadin et al., 1975).

WBR closely resembles vascular budding, a spontaneous formation of new buds from the vessels of the vascular system, first described in botryllid ascidians more than 200 years ago (Savigny, 1816) and observed and described again by Giard (1872); Bancroft (1903) and Herdman (1925). Vascular budding of botryllid ascidians is frequently associated with the process of estivation or hibernation (e.g., in *Botrylloides leachii*), during which colonies resorb their zooids to overcome adverse periods and reform their zooids from the tunic vessels when environmental conditions turn milder (Bancroft, 1903; Oka and Watanabe, 1959; Burighel et al., 1976; Atsumi and Saito, 2011). In *Botryllus primigenus*, *Botrylloides leni and Botryllus delicates*, vascular budding occurs continuously near the leading edge of the colony, at the bases of the ampullae (the blind endings of the tunic vessels), ensuring a quick increase in the size of the colony itself (Oka and Watanabe, 1957; Saito and Watanabe, 1985; Okuyama and Saito, 2001). Vascular budding has also been reported in the stolidobranch styelid *Symplegma brakenhielmi* (Gutierrez and Brown, 2017) and the phlebobranch *Perophora viridis* (Freeman, 1964).

In both WBR and vascular budding, hemocytes adhering to the vessel epithelium show the characteristics of stem cells, such as small size and large, round, euchromatic nuclei (Oka and Watanabe, 1957; Freeman, 1964; Rinkevich et al., 2007a, 2008), and are able to generate both the soma and germ line (Sunanaga et al., 2006). In the course of bud development, these cell aggregates grow and organize themselves to form the double vesicle stage, critical for bud organogenesis (Rinkevich et al., 1995; Oka and Watanabe, 1957; Voskoboynik et al., 2007). This characteristic stage is considered *a triploblastic vesicle of the gastrula type* (Brien, 1968), based on its organogenetic capacities: the outer vesicle is formed by the epidermis and will give rise to the zooid epidermis, whereas the inner vesicle and the intermediate mesenchyme cells will form all the internal tissues of the zooid (Manni and Burighel, 2006; Manni et al., 2007; Ricci et al., 2016).

WBR has been particularly well studied in *B. leachii*. In this species, the process occurs in five stages (Zondag et al., 2016; Blanchoud et al., 2017). In the first, lasting 15 h, wound healing is followed by a restructuring of the vessel architecture and of the ampullae, leading to the formation of small regeneration niches (stage 2). The contraction of the tissues marks stage 3, while homing of stem cells to the regeneration niches characterizes stage 4. Finally, competition among the various stem cell aggregates (stage 5) leads to the maturation of a single bud per experimental fragment (Rinkevich et al., 2007a,b, 2008; Zondag et al., 2016; Blanchoud et al., 2017).

The process of zooid formation from buds separated from the parental zooid in *Polyandrocarpa misakiensis* is considered analogous to WBR of botryllid ascidians (Kawamura et al., 2018). Here, buds are formed by the epidermis, the peribranchial epithelium and the mesenchyme cells between them. The situation resembles the double vesicle stage of botryllid ascidians and requires transdifferentiation of the peribranchial epithelium (Kawamura and Fujiwara, 1994, 1995).

As regards “stemness” markers, hemocyte aggregates do not express *piwi* in *B. primigenus* vascular buds (Sunanaga et al., 2010). However, hemocytes lining the vessel epithelium with the capacity to proliferate and expressing *piwi* have been postulated to play a role in the formation of the bud primordia in *Botrylloides violaceus* (Brown et al., 2009) and *B. leachii* (Rinkevich et al., 2010) WBR, as well as in *B. schlosseri* vascular budding. In *B. violaceus* WBR, *piwi*-positive hemocytes around the regenerating mass of cells are frequently immunolabeled by anti-PCNA antibodies: they have been hypothesized to be precursor cells that will be integrated into the developing bud as they start to differentiate (Brown et al., 2009).

Budding in *Botrylloides* WBR requires the presence of retinoid acid (RA), as inhibitors of RA synthesis block the process, whereas RA agonists accelerate bud formation and increase the number of buds per experimental fragment (Rinkevich et al., 2007b). Serine protease inhibitors alter the development of regeneration buds in *Botrylloides* (Rinkevich et al., 2007b), probably due to the role of serine proteases in remodeling the ECM, which is required for proper cell-cell communication during regeneration (Rinkevich et al., 2007a). This agrees with the observed increase in transcription of a trypsin-like serine protease upon RA treatment in the budding ascidian *P. misakiensis* (Ohashi et al., 1999). In *B. leachii*, the transcripts for aldehyde dehydrogenase, the enzyme involved in RA synthesis, and a serine protease similar to the mammalian urokinase-type plasminogen activator, are located in circulating phagocytes (Rinkevich et al., 2007a,b). This suggests a key role of these cells in the control of vascular budding and WBR, in addition to their ascertained role in palleal budding. *B. schlosseri* phagocytes are, in fact, required for proper clearance of apoptotic cells and corpses from the tissues of old zooids during the generation change. The recycling of nutrients derived from their digestion is required to support bud growth, as colonies are unable to feed during this period. This important phagocytic role is further supported by the observation that blocking phagocyte activity results in the arrest of blastogenesis (Voskoboynik et al., 2004). This also implies the involvement of innate immune responses, since phagocytes are key players in morphogenetic events of compound ascidians (Franchi and Ballarin, 2017). In accordance with this, *B. leachii* WBR is associated with the differential transcription of various immune-related genes (Rinkevich et al., 2007a, 2008).

## Concluding Remarks and Perspectives

Reviewing regeneration with a focus only on the contribution of cell proliferation, blastema formation, or totipotent ASCs leaves a large number of unanswered questions on the cellular and molecular underpinnings of this complex process. The impressive variety of regenerative mechanisms displayed within the animal kingdom makes it clear that adopting a comparative approach is as valuable as investigating emerging models. Importantly, the study of this fundamental biological phenomenon in invertebrate models can improve our understanding of core events in both regeneration-competent animals and those with reduced regenerative ability, such as humans. The vertebrate species most easily reared in captivity and used for regeneration studies -such as rodents, chickens, frogs or zebrafish- are costly to maintain, may possess quite limited regenerative abilities in adulthood, and their management is often problematic for ethical reasons. Many invertebrate deuterostomes, instead, show extensive adult regeneration, are easy to maintain in laboratory conditions, and, except in cases where they have protected status, their use for experimentation generally faces fewer restrictions. Past limitations, such as the availability of -omics data and techniques for genetic manipulation, are also rapidly disappearing. However, despite being reliable research organisms, they are still largely neglected as models in regeneration research.

Adult regeneration involves not only stem cell recruitment, but also dedifferentiation phenomena, which implies remarkable cellular plasticity. In deuterostomes, a large diversity of processes and cytotypes is often detectable even within the same phylum. Nevertheless, recruitment of cells deriving from the dedifferentiation of adult cells, rather than the use of resident stem cells, appears to predominate: indeed, in most clades, localized (tissue-specific) “recycling” of specialized cells is likely to occur. These cells generally originate from nearby tissues, which are locally remodeled and become the source of new cellular material. In most cases, such progenitors give rise to restricted types of cells, i.e., cells of each tissue regenerate elements of that tissue. There are, however, some exceptions, such as the multipotent epithelia of tunicates (which originate almost all tissues), and the coelomic epithelia of echinoderms (which generate the coelomocytes, the muscles and, likely, the skeleton). More such examples are likely to be discovered as research in this area intensifies with broader taxon sampling.

The involvement of resident undifferentiated cells during regeneration is generally limited, with the exception of undifferentiated amebocytes and coelomocytes in crinoids and hemoblasts in tunicates and, likely, of hemichordates and amphioxus. When present, these cells show a wide range of potency and, usually, are multi- or pluripotent stem cells or progenitor cells (Table 1).

**TABLE 1:** Main undifferentiated and differentiated cytotypes involved in invertebrate deuterostome regeneration.

Phylum	Clade	Phenomenon	Progenitor cells	Cells undergoing dedifferentiation
Echinodermata	Crinoidea	Arm and visceral mass regeneration	Amebocytes, coelomocytes	Muscle cells, coelomic epithelium, neurosecretory cells
	Echinoidea	Spine and test regeneration -	-	Muscle cells, sclerocytes
	Asteroidea	Arm regeneration	Coelomocytes	Muscle cells, coelomic epithelium
	Ophiuroidea	-	-	Muscle cells, coelomic epithelium
	Holothuroidea	-	-	Muscle cells, coelomic epithelium, glial cells
Hemichordata	Enteropneusta	Anterior and posterior regeneration	Circulating stem cells associated with blood vessels (?), mesenchymal cells (?)	Unclear
Cephalochordata	Leptocardii	Tail regeneration	Muscle satellite-like cells (?) in the tail; coelomocytes (?), mesenchymal cells (?) in oral cirri	Muscle cells (?), nerve cord cells (?), notochord cells (?) in the tail; skeletal rod cells in oral cirri (?)
Tunicata	Ascidiacea	Short distance partial body regeneration Long distance partial body regeneration Blastogenetic regeneration CCS regeneration WBR	ASCs in the oral siphon of *Ciona*, Cells from the peripharyngeal stem cell niches Bud tissues Vascular epithelium Hemoblasts	Bud tissues Vascular Epithelium Hemoblasts

***Terms and Definitions:** Blastema, Localized pool of cells, undifferentiated/pluripotent as well as retaining their tissue origin, usually of mesenchymal origin and enveloped by an epithelial layer, able to massively proliferate and differentiate into different cytotypes; Dedifferentiation, Process of cellular reprogramming by which differentiated (mature) somatic cells lose their specialization (oligo/unipotency) and revert to a less differentiated state (pluri/multipotency); Deuterostomes, Eumetazoans that during embryonic development show radial indeterminate cleavage and enterocoely, with the blastopore becoming the anus; Epithelial-Mesenchymal Transition, Dynamic and finely regulated process during which epithelial cells lose their epithelial features, disrupt their underlying basement membrane and assume features typical of mesenchymal cells, such as migratory capacity and production of extracellular matrix components. It is typical of embryonic development, tissue regeneration and morphogenesis, cancer invasion, wound healing, immune response, etc; Epimorphosis, Regeneration model mainly based on the proliferation and differentiation of cells composing the localized blastema; Morphallaxis, Regeneration model mainly based on the remodeling of pre-existing tissues without the formation of a localized blastema through significant local cell proliferation. It may involve the dedifferentiation of localized tissues or migration of cells from other distant locations; Progenitor Cells, Stem cell-derived cells lacking unlimited self-renewal capacity, and that differentiate into limited specialized cytotypes; Transdifferentiation, Process of cellular reprograming by which differentiated (mature) somatic cells convert directly into another type of differentiated (mature) somatic cell without passing through an intermediate pluripotent/multipotent state.*

The use of dedifferentiation as a major mechanism for tissue repair underscores the idea that cell plasticity in invertebrate deuterostomes is higher compared to vertebrates (Table 1). Among chordates, amphioxus and solitary ascidians, which show considerable but still relatively limited regeneration compared to other invertebrate deuterostome groups, can be considered good “transition” species between ambulacrarians and vertebrates. Conversely, the high regenerative ability of colonial ascidians has probably appeared secondarily, in association with asexual reproduction. This strict connection between regeneration and asexual reproduction is also true for echinoderms and hemichordates and is supported by the common molecular events associated with the two processes (Sánchez Alvarado, 2000). Although each tissue generally regenerates independently, it nevertheless has to do so in synchrony with the others to successfully restore a precise and accurate body plan. In this sense, regeneration is a very complex process that implies a precise and integrated response requiring the coordination of a plethora of different tissues, molecules and signals. Circulating cells can facilitate regeneration, especially in the first phases after injury, since they are overcoming tissue separation and allow both cell and signal spreading (Table 1). EMT and cell migration contribute to this general coordination.

Overall, it is difficult to strictly define the “stemness” properties of the cells involved in adult regeneration of invertebrate deuterostomes. In vertebrates, stem cells are usually characterized by precise undifferentiated ultrastructural features and specific molecular markers for pluri/multipotency, and may be present in well-defined niches. In contrast, in the invertebrate models reviewed here, cells without obvious localization in defined niches [with the possible exception of the lymph nodes (Ermak, 1976) and the endostyle in tunicates (Voskoboynik et al., 2008)] and with both undifferentiated and differentiated ultrastructural characters effectively act as stem cells, irrespective of their tissue of origin or the mechanisms by which they are recruited to the regeneration zone. Molecular data also underscore the important contribution of both undifferentiated and differentiated cells, although labeling and detailed cellular tracking are still required to pinpoint the precise origin and fate of these cells. Therefore, we propose a wider interpretation of the stem cell concept, not necessarily and strictly related to the classic idea of stem cells as undifferentiated cells, but including also cells deriving from dedifferentiation phenomena. This is in line with the definition of stem cells recently proposed by Post and Clevers (2019), which is based on their function rather than any morphological or molecular criteria: the ability to replace lost cells through cell division.

From an evolutionary viewpoint, the extensive cell plasticity described in adult invertebrate deuterostomes -both in terms of cell-lineage restriction and cell potency- may be one of the key elements of their successful regenerative responses. However, the relatively limited and patchy information available in these animal models renders difficult the identification of shared molecular pathways underpinning cell dedifferentiation, if indeed any exist. The observation that conserved developmental regulators -such as *Wnt*, *Notch* and *TGFβ/BMP*, to name just a few- are often re-deployed during regeneration may be less consistent with their roles as causative agents in the dedifferentiation/cell fate process, and more indicative of researcher prejudices for “favorite genes” and their ease of identification in homology-based searches. The question of which molecular switch might facilitate dedifferentiation in many invertebrates upon injury, and how this machinery may differ or be inactivated in non-regenerating organisms, is far more difficult to tackle and may lie rather in how epigenetic states and chromatin are regulated (Merrell and Stanger, 2016; Hayashi et al., 2020; Lee et al., 2020), or even in physical properties of the cellular microenvironment (Vining and Mooney, 2017). Further study of the various mechanisms regulating cell recruitment, dedifferentiation, and specification will help elucidate whether any are conserved between invertebrate and vertebrate deuterostomes, or rather represent a diverse and plastic repertoire of solutions to a common evolutionary problem.

Another intriguing aspect of regeneration is its relationship with immunity (Abnave and Ghigo, 2019; Arenas Gómez et al., 2020): various results indicate that the evolution of the adaptive immune system in vertebrates is correlated with a progressive loss of regenerative capability from fish to mammals (Godwin, 2014; Godwin and Rosenthal, 2014). This phenomenon appears related to the persistence of the inflammatory response, which impairs or limits the regeneration process, and scarring at the site of injury (Merscher and Neff, 2005; Eming et al., 2009; Godwin, 2014; Godwin and Rosenthal, 2014; Peiris et al., 2014; Fumagalli et al., 2018). In contrast, many invertebrates -including the highly regenerative models described here- show scar-free wound healing and yet have spectacularly complex and diverse innate immune repertoires (Rinkevich et al., 2007a; Huang et al., 2008; Ramírez-Gómez and García-Arrarás, 2010; Smith et al., 2010; Dishaw et al., 2012; Tassia et al., 2017; Ferrario et al., 2018; Oren et al., 2019), which have rapidly and often independently evolved to deal with pathogen exposure in a sort of host defense/pathogen arms race (Ghosh et al., 2011). However, the building blocks of RAG V(D)J recombinase activity on which vertebrate adaptive immunity relies have been found in invertebrate deuterostomes (Morales-Poole et al., 2017). Evidence is even emerging of immune system “memory” as part of a continuum between innate and acquired immunity (Milutinović and Kurtz, 2016; Netea et al., 2019). Therefore, the lack of adaptive immunity in invertebrate deuterostomes can partly explain their high regenerative potential, but is not entirely satisfactory when attempting to define causality in the face of such complexity. Our understanding of the impact of the immune response on healing and regeneration in most systems is at best rudimentary, and detailed studies of the consequences of the appearance of adaptive immunity for regeneration abilities are still lacking.

A continuing challenge in stem cell research is unequivocally distinguishing resident undifferentiated stem cells from the cellular products of dedifferentiation. In most non-model and emerging systems, including the invertebrate deuterostomes discussed here, differentiation state is inferred by mostly static snapshots of cells, accompanied by descriptions of limited molecular signatures or ultrastructural characteristics. Nevertheless, without such careful and detailed observations it is impossible to begin to elucidate the cellular basis of regeneration. The mechanisms regulating dedifferentiation are largely unknown, although research is beginning to identify dedifferentiation as a key player in a number of regenerative processes in genetically tractable vertebrate systems (Pesaresi et al., 2019; Aires et al., 2020). Therefore, while we believe that the available body of evidence indicates an overarching central role for dedifferentiation in invertebrate deuterostomes, caution still must be exercised in interpreting findings. Likewise, evidence for cell migration in regenerative processes is mostly indirect, although widely assumed to occur. Exceptions include a few studies in solitary and colonial ascidians as well as echinoderms (Voskoboynik et al., 2008; Jeffery, 2015b; Mashanov V. et al., 2017), where cells were labeled with lipophilic dyes to track cell movements. BrdU and EdU pulse/chase labeling of proliferating cells also give insight into not only division dynamics but also lineage and cell fate, particularly if fine control in labeling only a few cells is exercised. Comprehensive experiments are still lacking in many regenerating animals (but see Mashanov et al., 2013; Jeffery, 2015b; Cary et al., 2019), and are somewhat limited in scope, but do offer the advantage of not requiring extensive resources, and labeling is not affected by the changes in transcriptional or epigenetic state that occur during reprograming. Stable transgenic animals, or genetic labeling with inducible systems, permit by far the best control and resolution, and will be required to truly show changes in differentiation state and potency. Currently, transgenesis has been developed in some sea urchins (Buckley et al., 2018), amphioxus (Kozmikova and Kozmik, 2015), and the direct developing hemichordate *Saccoglossus kowalevskii* (Minor et al., 2019). A considerable molecular and genetic toolkit is also available for solitary ascidians, predominantly *C. intestinalis* but also *Phallusia mammillata* (Cota, 2018). However, transgenics have only been used to a limited degree, in *Ciona*, to study regeneration of the nervous system (Dahlberg et al., 2009), but hold great promise for elucidating the genetic basis of dedifferentiation, for instance during myocardium regeneration (Evans Anderson and Christiaen, 2016). The reasons for this lacuna are manifold. First, many invertebrate deuterostomes have long generation times, and rearing the larvae through metamorphosis to breeding age can be difficult. In addition, unlike models such as *Drosophila melanogaster* or *Danio rerio*, there are currently no repositories of genetic lines for any of the regenerating systems. This is in part due to the cost to maintain such centralized resources, but also to barriers to transport and importation of unusual (often marine) organisms. This is compounded by the sheer diversity of models being used for regeneration studies and the difficulty in acquiring funding for basic research on “weird animals”. However, sharing of knowledge and resources across the stem cell community, facilitated by initiatives such as the EU Horizon 2020 COST Action 16203 “MARISTEM” (Ballarin et al., 2018) will pave the way for more rapid uptake and development of the tools required to answer fundamental questions about stem cell biology in our closest living relatives.

In conclusion, the greatest challenge for the regeneration field, from the perspective of future human medical applications, is to compare invertebrate and vertebrate deuterostomes effectively, in terms of cells and mechanisms involved in the regenerative process. The identification of potential commonalities as well as differences will be crucial to the goal to improve the rather limited regenerative capabilities in humans. Cell tracking, coupled with molecular and microscopy approaches, will be critical to address some of the main issues in animal regeneration, i.e., the understanding of the origin and fate of recruited cells. As such, it is first necessary to characterize different cytotypes and identify cell-specific molecular markers in order to visualize and recognize the cells involved in regeneration.

Next generation technologies, from single cell to ChIP-sequencing and proteomics, in combination with novel bioinformatic platforms and statistical analyses, will be instrumental in achieving this objective. The development of *in vitro* systems, still very difficult in invertebrate deuterostomes (Di Benedetto et al., 2014b; Mercurio et al., 2014), will provide a further tool to investigate these problems. Ultimately, researchers need to ask the right questions and identify the model animals most appropriate for the study of regeneration. Only with a solid investment in understanding the diversity of cellular mechanisms underlying the remarkable regenerative ability seen in invertebrate models can we hope to unlock the dormant potential of vertebrate systems.

## Author Contributions

CF, MS, IMLS, and LB conceived the work and wrote the manuscript. All authors contributed to the article and approved the submitted version.

## Conflict of Interest

The authors declare that the research was conducted in the absence of any commercial or financial relationships that could be construed as a potential conflict of interest.
